# A pilot study to explore patterns and predictors of delayed kidney decline after cardiopulmonary bypass

**DOI:** 10.1038/s41598-024-57079-x

**Published:** 2024-03-20

**Authors:** Ahmed Zaky, Duraid S. Younan, Bradley Meers, David Miller, Ryan L. Melvin, David Benz, James Davies, Brent Kidd, Mali Mathru, Ashita Tolwani

**Affiliations:** 1 Department of Anesthesiology and Critical Care Medicine, University of Alabama at Birmingham, 950 Jefferson Tower, 625 19th Street South, Birmingham, AL 35249-6810 USA; 2 Department of Surgery, Staten Island University, Staten Island, USA; 3 Department of Cardiothoracic Surgery, University of Alabama at Birmingham, Birmingham, USA; 4 Division of Critical Care, Department of Anesthesiology, University of Kansas Medical Center, Kansas City, USA; 5 Department of Nephrology, University of Alabama at Birmingham, Birmingham, USA

**Keywords:** Cardiology, Diseases

## Abstract

There is no current consensus on the follow up of kidney function in patients undergoing cardiopulmonary bypass (CPB). The main objectives of this pilot study is to collect preliminary data on kidney function decline encountered on the first postoperative visit of patients who have had CPB and to identify predictors of kidney function decline post hospital discharge. Design: Retrospective chart review. Adult patients undergoing open heart procedures utilizing CPB. Patient demographics, type of procedure, pre-, intra-, and postoperative clinical, hemodynamic echocardiographic, and laboratory data were abstracted from electronic medical records. Acute kidney disease (AKD), and chronic kidney disease (CKD) were diagnosed based on standardized criteria. Interval change in medications, hospital admissions, and exposure to contrast, from hospital discharge till first postoperative visit were collected. AKD, and CKD as defined by standardized criteria on first postoperative visit. 83 patients were available for analysis. AKD occurred in 27 (54%) of 50 patients and CKD developed in 12 (42%) out of 28 patients. Older age was associated with the development of both AKD and CKD. Reduction in right ventricular cardiac output at baseline was associated with AKD (OR: 0.5, 95% CI: 0.3, 0.79, P = 0.01). Prolongation of transmitral early diastolic filling wave deceleration time was associated with CKD (OR: 1.02, 95% CI: 1.01, 1.05, P = 0.03). In-hospital acute kidney injury (AKI) was a predictor of neither AKD nor CKD. AKD and CKD occur after CPB and may not be predicted by in-hospital AKI. Older age, right ventricular dysfunction and diastolic dysfunction are important disease predictors. An adequately powered longitudinal study is underway to study more sensitive predictors of delayed forms of kidney decline after CPB.

## Introduction

Cardiac surgery requiring cardiopulmonary bypass (CPB) is a common cause of acute kidney injury (AKI). Even milder forms of AKI may progress to chronic kidney disease (CKD) and are associated with increased mortality and morbidity^1,2^.

The Kidney Disease Improving Global Outcomes (KDIGO) defines AKI as an abrupt decrease in kidney function that occurs over the course of 7 days or less and CKD as abnormalities in kidney structure that persist over > 90 days. The term acute kidney disease (AKD) has been proposed to define ongoing processes that bridge the progression of AKI to CKD. AKD is defined as acute or subacute damage and/or loss of kidney structure for a duration > 7 days yet < 90 days after an AKI precipitating event^3^.

Caveats exist in the current KDIGO criteria that may influence the postoperative course and care of patients undergoing CPB. For example, patients may not manifest an AKI during their hospital course yet present with a kidney function decline at the first follow up visit. In such an instance, it is unknown whether these patients manifest a new onset AKI or an AKD from an ongoing injury that has not manifested during hospital discharge. Furthermore, waiting for 90 days to fulfill criteria for CKD may expose these patients to further irreversible kidney damage. The picture becomes more confusing in the absence of a consensus on the follow up of patients who do not manifest in-hospital AKI.

To remedy this gap in the less studied delayed forms of kidney decline post CPB^4–6^, we report our preliminary data on kidney function at the first postoperative visit in a cohort of our patients who have undergone CPB^7^. Our secondary objective is to assess predictors of kidney function decline at first postoperative visit.

## Discussion

In this pilot study we demonstrate a delayed pattern of postoperative kidney dysfunction that is not predicted by baseline kidney function nor by postoperative acute kidney injury and that is not explained by factors known to precipitate a new onset kidney injury. Age is a common predictor of AKD and CKD. Right ventricular (RV) dysfunction is a predictor of AKD. Prolonged deceleration time is a predictor of CKD.

Our study is in alignment with other studies that demonstrate a continuum of kidney decline in the cardiac surgical setting^8–13^. Matsuura et al.^13^ published a study demonstrating an association between AKD and 90-day mortality and 2-year decline in eGFR. Compared to our study, Matsuura et al.’s was a much larger study and studied outcomes over a longer period of observation. Our study, on the other hand, reported AKD and CKD as outcomes and reported predictors on their association Compared with the study by Legouis et al.^4^, our study did not demonstrate and association of AKD and CKD with postoperative AKI. This contradiction might be attributed to the more patients with advanced stages of AKI in Legouis et al. study compared to ours. Also we measured eGFR as a change from baseline compared to Legouis et al.’s study that defined CKD based on an absolute reduction in eGFR^4^ below 60 mL/min/1.73 m^2^. Our study is in line with others that emphasized age as an independent risk factor for AKI postoperatively^14–16^. Whereas age is an important predictor of AKI, its role in more chronic forms of kidney disease is debatable^11,17^. The ‘natural’ decline of eGFR with aging may overestimate the incidence of CKD or progressive kidney disease occurring in this population. Our study contradicts with other studies that demonstrate age-adjusted eGFR is not associated with progression, or even may be associated with regression of CKD^18,19^. In contradistinction of these population studies that followed medical volunteers, our study was performed on surgical patients within the realm of an acute insult to the kidneys. We speculate that the non-physiologic state of CPB might have accentuated a steady ongoing nephron loss process occurring in the elderly. As such, an acute insult may overcome the organ reserve of the elderly leading to renal functional deterioration^20^. Whether this decline in eGFR is a progressive versus a regressive, remains to be proven. This study is in line with a larger study by the same principal investigator that demonstrated an incidence of 20% of postoperative long-term kidney decline in patients without postoperative AKI undergoing endovascular aortic aneurysm repair^21^. Despite a comparatively higher proportion of patients with postoperative long-term kidney decline who developed postoperative AKI, a 20% is not an insignificant percent. Despite the dissimilarity in the surgical setting, the similarities in the surgical population between cardiac and vascular surgical population warrant the search for more sensitive markers that can differentiate acute from chronic kidney disease. It is important to highlight that we did not stratify eGFR and that we did not follow up these patients to determine the course of disease progression. Equally important, it remains to be determined how elderly patients who are at risk of being impacted by CPB in terms of comorbidities, intraoperative hemodynamics and other factors that affect renal function under CPB^22^.

The absence of a new insult known to precipitate AKI leads us to speculate a subacute ongoing mechanism of kidney dysfunction commencing with surgery that is unrecognized by insensitive markers of AKI currently in use. The association of kidney disease with a reduction in RV CO and with prolonged deceleration time with the latter being a marker of diastolic dysfunction, proposes a forward hypoperfusion and backward congestive mechanisms to kidney disease, respectively. Important to appreciate that our study was not adequately powered to study predictors of kidney decline. Therefore, these findings remain hypothesis generating at the current stage.

Our study is in line with others that recognize AKD after cardiac surgery^13^. Yet, we differ than those that demonstrated AKD as an extension of AKI or of baseline CKD.

Small sample size and lack of long-term follow up stand as limitations of our study. The small sample size represents real world circumstances where there is no consensus on following up of renal function post hospital discharge. Furthermore, lack of data on urine output and proteinuria may have altered the results. Yet, this might have resulted in diagnosing more cases with kidney function decline.

In summary, this exploratory study demonstrates that CBP-requiring cardiac surgery is associated with delayed patterns of kidney function decline. As such, these patterns, while may deviate from current standardized definitions, are important potential therapeutic targets. An adequately powered longitudinal study is under way to elucidate more sensitive in-hospital predictors of delayed kidney function decline in patients undergoing CPB.

## Results

**Figure 1:**
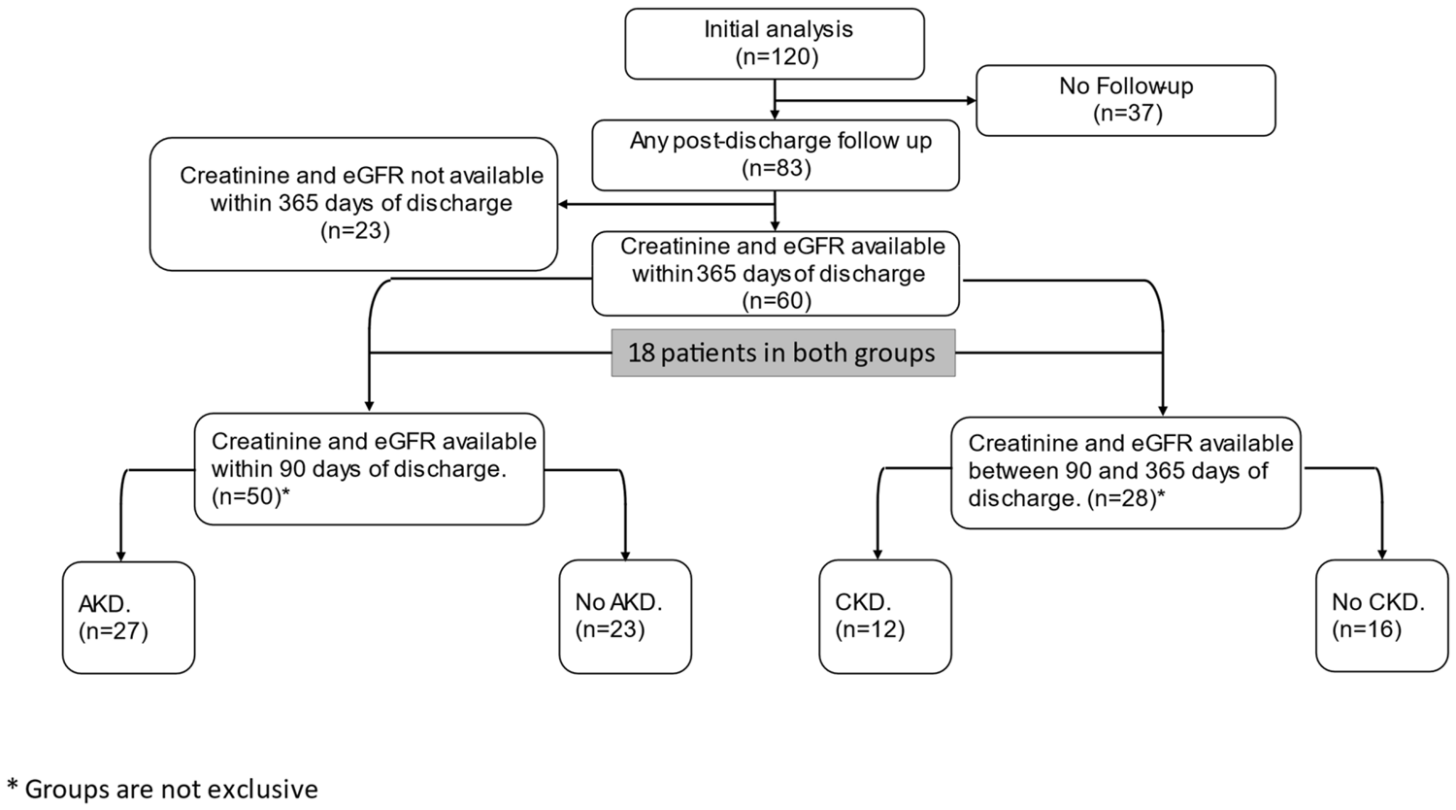
Study flow chart.

**Figure 2:**
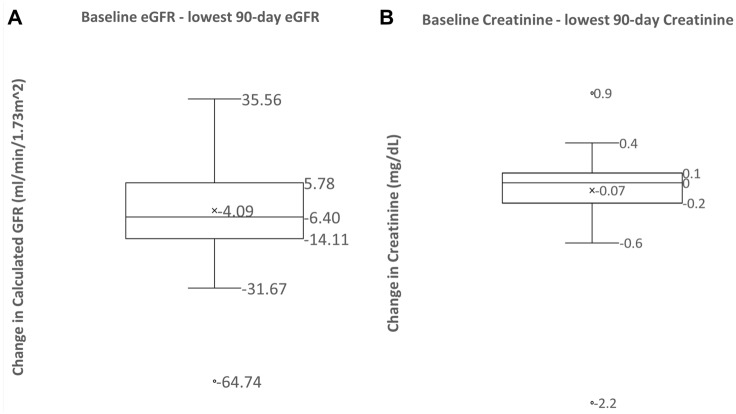
(**A**) A box-and-whisker plots conveys the distribution of 90-day eGFR changes (defined as the difference between the lowest eGFR measurement within 90 days of discharge and the baseline eGFR). The average change is − 4.09 mL/min/1.73 m^2^. The median change is − 6.40 mL/min/1.73 m^2^. (**B**) A box-and-whisker plots conveys the distribution of 90-day creatinine changes (defined as the difference between the lowest creatinine measurement within 90 days of discharge and the baseline creatinine). The average change is − 0.07 mg/dL. The median change is 0.10 mg/dL.

**Figure 3:**
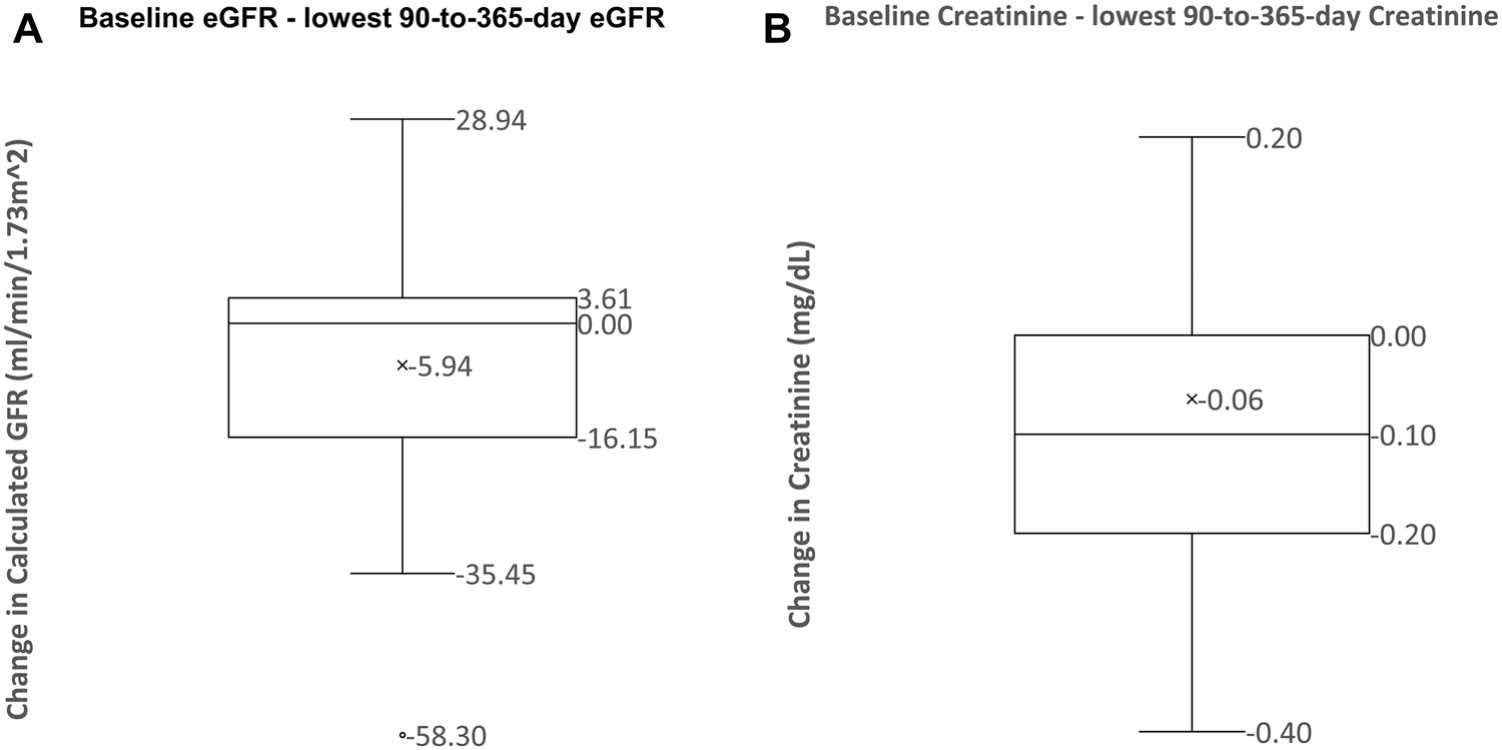
(**A**) A box-and-whisker plots conveys the distribution of 91-to-365-day eGFR changes (defined as the difference between the lowest eGFR measurement between 91 and 365 days of discharge and the baseline eGFR). The average change is − 5.94 mL/min/1.73 m^2^. The median change is 0 mL/min/1.73 m^2^. (**B**) A box-and-whisker plots conveys the distribution of 91-to-365-day creatinine changes (defined as the difference between the lowest creatinine measurement between 91 and 365 days of discharge and the baseline creatinine). The average change is − 0.06 mg/dL. The median change is 0 mg/dL.

The original cohort consisted of 120 patients who were analyzed for volume responsiveness^7^. Of these, 36 patients (28%) developed AKI. Of the original 120 patients only 83 patients had postoperative serum creatinine or eGFR value recorded after hospital discharge (Fig. 1). Characteristics of patients who developed and those who did not develop AKD, and CKD are summarized in Table 1. Older age was associated with the development of AKD and CKD. Reduction in eGFR from baseline averaged 4 mL/min/1.73 m^2^ and 6 mL/min/1.73 m^2^ in the first 90 days (Fig. 2) and between 91–365 days, respectively (Fig. 3).

**Table 1:** Demographics, preoperative, and follow-up variables.

Variable	90 day decline (n = 27)	No 90 day decline (n = 23)	365 day decline (n = 12)	No 365 day decline (n = 16)
***Age***	65.04	57.39*	63.33	52.19*
Gender—female	10	7	6	7
BMI	31.67	29.1	30.54	31.47
Race—Black or African American	6	6	2	1
Race—White	21	15	10	15
Race—Hispanic or Latino	0	1	0	0
Race—Asian	0	1	0	0
CKD—No	20	19	10	13
CKD—Yes	7	4	2	3
Baseline eGFR	80.56	74.78	79.19	84.92
AKI—No	20	18	11	16
AKI—Yes	7	5	1	0
AKI_Stage—None	20	18	9	11
AKI_Stage—Stage Yes	7	4	3	5
AKI_Stage—Stage 2	0	1	0	0
COPD—No	25	15	10	11
COPD—Yes	1	5	1	1
Hypertension—Yes	2	6	3	4
BetaBlocker—Yes	8	7	4	2
ACEI—Yes	4	3	1	0
ACE_Inhibitors_atfirstGFR—Yes	0	2	1	0
ARBs_atfirstGFR—Yes	1	1	0	0
NSAIDS_atfirstGFR—Yes	2	0	1	1
Antidiabetics_atfirstGFR—Yes	3	3	2	0
Antibiotics_atfirstGFR—Yes	1	0	0	0
EuroScore	4.79	6.89	6.09	4.13

*P < 0.05 denoting statistical significance. P-values are from Fisher’s Exact Test (COPD, CKD, Diabetes, Hypertension, Gender, Race, Beta blocker, ACEI, AKI, Medications at follow up) or from two-sample *t*-test (age, BMI, EuroScore, and Baseline GFR). These continuous variables have group means displayed rather than counts. Names of variables with statistically significant differences (P < 0.05) are emphasized with bold and italics. Note that differences between category totals and group totals are due to missing data. A 90-day decline is defined as a negative value yielded by the lowest eGFR measurement within 90 days of discharge subtracted from the baseline eGFR. A 365-day decline is defined as a negative value yielded by the lowest eGFR measurement within 91 to 365 days of discharge subtracted from the baseline eGFR. *ACEI* angiotensin converting enzyme inhibitors, *COPD* chronic obstructive pulmonary disease, *CKD* chronic kidney disease, *BMI* body mass index, *eGFR* estimated glomerular filtration rate, *ARBs* angiotensin receptor blockers, *NASIDs* non-steroidal anti-inflammatory drugs.

No significant differences existed between those who developed and those who did not develop either of AKD and CKD in terms of preoperative eGFR, exposure to medications known to affect perioperative kidney function, the distribution of comorbidities, or average severity scores during hospital stay, average serum lactate for their hospital stay, cross clamp time and duration on CPB. Coronary artery bypass grafting was the most frequent procedure with no difference between those who developed and those who did not develop AKD and CKD (data not shown). There were no significant differences between those who developed and those who did not develop each of AKD and CKD in the incidence of postoperative AKI (Supplementary Tables 1, 2).

Multi-stage logistic regression demonstrated age and increased right ventricular cardiac output prior to institution of CPB to independently predict AKD. On the other hand, age and prolonged deceleration time were independently associated with CKD (Tables 2 and 3). Deceleration time refers to the time taken form the peak velocity of the early filling waveform (E wave) to baseline as measure by spectral Doppler^23^.

**Table 2:** 90-day decline single-variable and multivariable logistic regression results.

Variable	P	Odd ratio (OR)	95% confidence interval (CI)	Adjusted P	Adjusted OR	Adjusted CI
***RVOT_CO_1***	0.01	0.51	(0.3, 0.79)	0.02	0.42	(0.17, 0.78)
***Age***	0.04	1.05	(1, 1.11)	0.02	1.10	(1.02, 1.22)

Univariable logistic regression models were used to investigate the association between each covariate and eGFR decline within 90 days after discharge, compared to baseline. P-values, odds ratios, and confidence intervals are reported in the 2nd, 3rd, and 4th columns respectively. Any covariate that was significant at P < 0.05 in a univariable model was then entered into a multivariable logistic regression model. The adjusted P-values, odds ratios, and confidence intervals are reported in the 5th, 6th, and 7th columns. Names of variables with statistically significant coefficients (P < 0.05) in the multivariable models are emphasized with bold and italics. A 90-day decline is defined as a negative value yielded by the lowest eGFR measurement within 90 days of discharge subtracted from the baseline eGFR. *RVOT-CO_1* cardiac output measured across the right ventricular outflow tract- pre cardiopulmonary bypass.

**Table 3:** 91-to-365-day decline single-variable and multivariable logistic regression results.

Variable	P	Odd ratio (OR)	95% confidence interval (CI)	Adjusted P	Adjusted OR	Adjusted CI
***DT_3***	0.02	1.02	(1.01, 1.05)	0.04	1.02	(1.01, 1.05)
Age	0.05	1.07	(1.01, 1.16)	0.25	1.08	(0.97, 1.28)

Univariable logistic regression models were used to investigate the association between each covariate and eGFR decline between 91 and 365 days after discharge, compared to baseline. P-values, odds ratios, and confidence intervals are reported in the 2nd, 3rd, and 4th columns respectively. Any covariate that was significant at P < 0.05 in a univariable model was then entered into a multivariable logistic regression model. The adjusted P-values, odds ratios, and confidence intervals are reported in the 5th, 6th, and 7th columns. Names of variables with statistically significant coefficients (P < 0.05) in the multivariable models are emphasized with bold and italics. A 365-day decline is defined as a negative value yielded by the lowest eGFR measurement within 91 to 365 days of discharge subtracted from the baseline eGFR. *DT-3* deceleration time measured at chest closure.

## Methods

This is a retrospective chart review. The study is an extension of our published retrospective study reporting on the association of end of procedure volume responsiveness and AKI^7^. The original study focused on patients who developed AKI during their hospital stay. Comparatively, the current study focuses on patients after their hospital discharge and on more delayed forms of kidney function decline after CPB-requiring procedures. The study was approved by the Institutional Review Board (IRB#150508005) of The University of Alabama At Birmingham (UAB) and adhered to the Declaration of Helsinki Informed written consent was obtained from all patients participating in this study in accordance with regulatory standards. Adult patients undergoing open heart procedures utilizing CPB were enrolled in the study. Patients < 19 years old, those with estimated glomerular filtration rate (eGFR) < 30 mL/kg/min^2^, transplanted patients, patients with preoperative ejection fraction < 30%, and patients undergoing mechanical assist device placement, off pump coronary artery bypass grafting or percutaneous valve interventions were excluded from the study. The authors complied with STROBE Guidelines on reporting cohort studies. A study flow chart is shown in Fig. 1.

### Data collection

Patient demographics, type of procedure, pre-, intra-, and postoperative clinical, hemodynamic echocardiographic, and laboratory data were routinely collected and abstracted from electronic medical records (EMR) for up to 7 days of hospital stay and at the first post discharge clinic visit. Acute kidney injury, and chronic kidney disease (CKD) were diagnosed based on KDIGO standardized criteria for serum creatinine and eGFR, respectively^24^. Intraoperative transesophageal echocardiography was performed pre- immediately post- CPB and at chest closure. Kidney function was assessed on the first postoperative visit up to 1 year and was stratified as within the first 90 days (acute kidney disease, AKD) versus 91–365 days post hospital discharge (chronic kidney disease, CKD). Interval change in medications, hospital admissions, and exposure to contrast, from hospital discharge till first postoperative visit were collected from EMR. Patients who were exposed to any of the aforementioned insults were excluded from the analysis.

### Outcomes

The primary outcome was development of AKD and CKD as defined by KDIGO standardized criteria for serum creatinine and eGFR on first postoperative visit^3^.

### Statistical analysis

Given the exploratory nature of the study, no power calculation was performed. For binary variables, Chi-squared test was used to investigate the association between each covariate and kidney disease. The Student’s *t*-test and the Wilcoxon rank sum test were used to compare independent continuous variables between a patient with and without kidney disease for normally and non- normally distributed variables, respectively. Univariable logistic regression models were used to investigate the association between each covariate and kidney disease. Any covariate that was significant at P < 0.05 was then entered into a multivariable model. Variables with high Variance Inflation Factor (VIF > 4) were removed to avoid collinearity. Additionally, variables with more than 25% missingness were excluded from the multivariate model. Logistic regressions, *t*-tests, and Pearson's Chi-squared tests with Yates’ continuity correction were conducted in R version 4.0.2.

### Institutional review board statement

The study was conducted in accordance with the Declaration of Helsinki, and approved by the Institutional Review Board of UNIVERSITY OF ALABAMA (protocol code #150508005, 9/25/2017).”

### Informed consent

Informed consent was obtained from all subjects involved in the study. Written informed consent has been obtained from all participating patients to publish this paper.

### Supplementary Information

Supplementary Tables.

## Data Availability

Data used and/or analyzed in this study will be available upon a reasonable request from the first author according to institutional guidelines for data sharing.

## Abbreviations

CS-KD: Cardiac surgery related kidney dysfunction
CPB: Cardiopulmonary bypass
IRB: Institutional Review Board
UAB: University of Alabama
EMR: Electronic medical records
AKD: Acute kidney disease
CKD: Chronic kidney disease
AKI: Acute kidney injury
KDIGO: Kidney Disease Improving Global Outcomes
VIF: Variance inflation factor
