# Supplementary material for: A pilot study to explore patterns and predictors of delayed kidney decline after cardiopulmonary bypass
Source: Sci Rep. 2024 Mar 20;14:6739. doi: 10.1038/s41598-024-57079-x (PMC10954642; doi:10.1038/s41598-024-57079-x)
Supplement: Supplementary file 1 — Supplementary Tables. [file 41598_2024_57079_MOESM1_ESM.docx]

**Supplementary tables**

**Supplementary Table A1*.* All variables considered for 90-day decline**

| **Variable** | **P** |
| --- | --- |
| AKI | 0.73 |
| LVEDV.1 | 0.34 |
| LVEDV.2 | 0.15 |
| LVEDV.3 | 0.07 |
| LV.ESV.1 | 0.21 |
| LV.ESV.2 | 0.10 |
| LV.ESV.3 | 0.07 |
| LV SV 1 | 0.36 |
| LVSV2 | 0.27 |
| LVSV3 | 0.25 |
| LVEF.1 | 0.20 |
| LVEF.2 | 0.30 |
| LVEF.3 | 0.47 |
| LV CO 1 | 0.29 |
| LV CO 2 | 0.17 |
| LV CO 3 | 0.37 |
| LV E 1 | 0.85 |
| LV E 2 | 0.08 |
| LV E 3 | 0.76 |
| LV A 1 | 0.19 |
| LV A 2 | 0.08 |
| LV A 3 | 0.46 |
| DT 1 | 0.29 |
| DT 2 | 0.11 |
| DT 3 | 0.27 |
| LV.E.A.1 | 0.20 |
| LV.E.A.3 | 0.23 |
| RVEDV 1 | 0.16 |
| RVEDV 2 | 0.11 |
| RVEDV 3 | 0.28 |
| RVESV 1 | 0.13 |
| RVESV 2 | 0.08 |
| RV ESV 3 | 0.15 |
| RVSV1 | 0.10 |
| RVSV2 | 0.11 |
| RVSV3 | 0.21 |
| RV EF 1 | 0.09 |
| RV EF 2 | 0.80 |
| RV EF 3 | 0.38 |
| TAPSE 1 | 0.14 |
| TAPSE 3 | 0.62 |
| RVOT CO 1 | 0.01 |
| RVOT CO 2 | 0.15 |
| RVOT CO 3 | 0.50 |
| RV E 1 | 0.64 |
| RV E 3 | 0.48 |
| RV A 1 | 0.99 |
| RV A 3 | 0.67 |
| RV DT 1 | 0.99 |
| RV DT 3 | 0.17 |
| RV E.A 1 | 0.41 |
| RV E.A 3 | 0.94 |
| HV D1 | 0.18 |
| HV D3 | 0.53 |
| HV S1 | 0.79 |
| HV S3 | 0.46 |
| HV AR 1 | 0.32 |
| HV AR 3 | 0.69 |
| HV S.D 1 | 0.10 |
| HV S.D 3 | 0.72 |
| ESV.SV 1 | 0.47 |
| heart rate 1.y | 0.31 |
| heart rate 2 | 0.08 |
| heart rate 3 | 0.14 |
| heart rate icu | 0.57 |
| pul sys blood pressure 1 | 0.36 |
| pul dia blood pressure 1 | 0.94 |
| pul mean blood pressure 1 | 0.52 |
| art sys blood pressure 1 | 0.63 |
| art dia blood pressure 1 | 0.63 |
| art mean blood pressure 1 | 0.32 |
| pul sys blood pressure 2 | 0.19 |
| pul dia blood pressure 2 | 0.14 |
| pul mean blood pressure 2 | 0.67 |
| art sys blood pressure 2 | 0.55 |
| art dia blood pressure 2 | 0.40 |
| art mean blood pressure 2 | 0.32 |
| pul sys blood pressure 3 | 0.83 |
| pul dia blood pressure 3 | 0.78 |
| pul mean blood pressure 3 | 0.52 |
| art sys blood pressure 3 | 0.97 |
| art dia blood pressure 3 | 0.79 |
| art mean blood pressure 3 | 0.36 |
| systolic blood pressure ic | 0.06 |
| diastolic blood pressure i | 0.87 |
| fio2 1 | 0.02 |
| fio2 2 | 0.76 |
| fio2 3 | 0.87 |
| fio2 icu | 0.30 |
| paco2 1 | 0.55 |
| paco2 2 | 0.17 |
| paco2 icu | 0.96 |
| temp 1 | 0.55 |
| temp 2 | 0.59 |
| temp 3 | 0.38 |
| temp icu | 0.11 |
| cvp 1 | 0.33 |
| cvp 2 | 0.44 |
| cvp 3 | 0.45 |
| pasp 1 | 0.36 |
| pasp 2 | 0.19 |
| pasp 3 | 0.83 |
| padp 1 | 0.94 |
| padp 2 | 0.14 |
| padp 3 | 0.78 |
| cross clamp time | 0.08 |
| cpb time | 0.19 |
| creat1 | 0.64 |
| or fluid in | 0.19 |
| or fluid out | 0.42 |
| or net balance | 0.74 |
| rbc 1 | 0.58 |
| plt 1 | 0.14 |
| plt 2 | 0.66 |
| egfr 1 | 0.80 |
| hematocrit 1 | 0.92 |
| postop hr 1 | 0.57 |
| postop mabp 1 | 0.43 |
| postop temp 1 | 0.11 |
| postop mvo2 1 | 0.61 |
| uop 1 | 0.80 |
| uop 2 | 0.76 |
| uop 3 | 0.61 |
| uop 4 | 0.05 |
| uop 5 | 0.45 |
| uop 6 | 0.60 |
| uop 7 | 0.17 |
| age | 0.04 |
| smoking | 0.88 |
| body weight | 0.21 |
| Gender Male | 0.62 |
| BMI | 0.22 |
| CKD | 0.47 |
| Anemia | 0.11 |
| Beta Blocker | 0.95 |
| ACEI | 0.86 |
| Lowest Hct | 0.95 |
| Race Black or African American | 0.99 |
| Race Hispanic or Latino | 1.00 |
| Race White | 0.99 |
| Surgical category - combined | 0.45 |
| Surgical category - other | 0.90 |
| Surgical category - Valve | 0.62 |
| Surgery redo | 0.87 |
| LVEF.PRE1.CATModerate | 0.78 |
| LVEF.PRE1.CATPoor | 0.21 |
| Pulmonary Hypertension | 0.06 |
| COPD | 0.06 |
| Unstable Angina | 0.99 |
| COPD Factor | 0.06 |
| Creatinine Factor | 0.99 |
| LV Factor | 0.38 |
| Procedure Other Than CABG | 0.80 |
| Thoracic Aorta | 0.99 |
| Euro Score | 0.21 |
| Baseline eGFR | 0.35 |
| SV UP | 0.67 |
| SV DOWN | 0.52 |
| volume percent change | 0.99 |
| volume responsive | 0.19 |
| AKI Stage Stage 1 | 0.52 |
| AKI Stage Stage 2 | 0.99 |
| ACE Inhibitors at first GFR | 0.99 |
| ARBs at first GFR | 0.91 |
| NSAIDS at first GFR | 0.99 |
| Antidiabetics at first GFR | 0.83 |
| Antibiotics at first GFR | 0.99 |
| log Euro Score | 0.29 |
| Previous Cardiac surgery | 0.87 |
| Hypertension | 0.06 |
| Mean 7-day postop Lactic Acid | 0.04 |
| LV EA1 ratio | 0.26 |
| LV EA2 ratio | 0.66 |
| LV EA3 ratio | 0.30 |

P-values are from single-variable logistic regression models with 90-day GFR decline as the dependent (y) variable. In the case of categorical variables, each category is reported separately, but each category was a binary variable in a single logistic regression. AKI: acute kidney injury; LV: left ventricle; EDV: end diastolic volume; ESV: end systolic volume; SV: stroke volume; EF: ejection fraction; RV: right ventricle, CO: cardiac output; E: early diastolic peak velocity; A: late diastolic peak velocity; E/A ration of early to late peak diastolic velocities; DT: deceleration time; TAPSE: tricuspid annular plane systolic excursion; FIO2: fractional inspired oxygen concentration; PASP: pulmonary artery systolic pressure, PADP; pulmonary artery diastolic pressure; UOP: urinary output; temp: temperature; PaCO2:arterial partial pressure of carbon dioxide; PaO2: arterial partial pressure of oxygen; ACEI: angiotensin converting enzyme inhibitors; COPD: chronic obstructive pulmonary disease; CKD: chronic kidney disease; BMI: body mass index; eGFR: estimated glomerular filtration rate; ARBs: angiotensin receptor blockers; NASIDs: non-steroidal anti-inflammatory drugs; CABG: coronary artery bypass grafting; COPD: chronic obstructive pulmonary disease; Hct: hematocrit; CKD: chronic kidney disease; CVP: central venous pressure; RBCS: red blood cells; plts: platelets; FFP; fresh frozen plasma; LVEF.PRE1.CATModerate: moderate reduction in ejection fraction(EF 40-65%); LVEF.PRE1.CATPoor; severe reduction in EF (EF < 40%), ICU; intensive care unit; SV UP/DOWN: stroke volume with head up and down, respectively; eGFR: estimated glomerular filtration rate; in all, numerals,: time points for measurements.

**Supplementary Table A2*.* All variables considered for 365-day decline**

| **Variable** | **P** |
| --- | --- |
| AKI | 0.72 |
| LVEDV.1 | 0.74 |
| LVEDV.2 | 0.66 |
| LVEDV.3 | 0.73 |
| LV.ESV.1 | 0.91 |
| LV.ESV.2 | 0.61 |
| LV.ESV.3 | 0.66 |
| LV SV 1 | 0.29 |
| LVSV2 | 0.79 |
| LVSV3 | 0.46 |
| LVEF.1 | 0.78 |
| LVEF.2 | 0.71 |
| LVEF.3 | 0.20 |
| LV CO 1 | 0.16 |
| LV CO 2 | 0.50 |
| LV CO 3 | 0.30 |
| LV E 1 | 0.94 |
| LV E 2 | 0.44 |
| LV E 3 | 0.95 |
| LV A 1 | 0.20 |
| LV A 2 | 0.08 |
| LV A 3 | 0.32 |
| DT 1 | 0.87 |
| DT 2 | 0.42 |
| DT 3 | 0.02 |
| LV.E.A.1 | 0.12 |
| LV.E.A.3 | 0.66 |
| RVEDV 1 | 0.07 |
| RVEDV 2 | 0.41 |
| RVEDV 3 | 0.81 |
| RVESV 1 | 0.09 |
| RVESV 2 | 0.56 |
| RV ESV 3 | 0.30 |
| RVSV1 | 0.47 |
| RVSV2 | 0.07 |
| RVSV3 | 0.22 |
| RV EF 1 | 0.79 |
| RV EF 2 | 0.75 |
| RV EF 3 | 0.54 |
| TAPSE 1 | 0.82 |
| TAPSE 3 | 0.91 |
| RVOT CO 1 | 0.68 |
| RVOT CO 2 | 0.45 |
| RVOT CO 3 | 0.77 |
| RV E 1 | 0.61 |
| RV E 3 | 0.08 |
| RV A 1 | 0.18 |
| RV A 3 | 0.49 |
| RV DT 1 | 0.14 |
| RV DT 3 | 0.94 |
| RV E.A 1 | 0.54 |
| RV E.A 3 | 0.25 |
| HV D1 | 0.85 |
| HV D3 | 0.77 |
| HV S1 | 0.81 |
| HV S3 | 0.91 |
| HV AR 1 | 0.53 |
| HV AR 3 | 0.20 |
| HV S.D 1 | 0.62 |
| HV S.D 3 | 0.93 |
| ESV.SV 1 | 0.06 |
| heart rate 1.y | 0.93 |
| heart rate 2 | 0.16 |
| heart rate 3 | 0.74 |
| heart rate icu | 0.58 |
| pul sys blood pressure 1 | 0.28 |
| pul dia blood pressure 1 | 0.43 |
| pul mean blood pressure 1 | 0.73 |
| art sys blood pressure 1 | 0.45 |
| art dia blood pressure 1 | 0.66 |
| art mean blood pressure 1 | 0.47 |
| pul sys blood pressure 2 | 0.24 |
| pul dia blood pressure 2 | 0.51 |
| pul mean blood pressure 2 | 0.47 |
| art sys blood pressure 2 | 0.24 |
| art dia blood pressure 2 | 0.70 |
| art mean blood pressure 2 | 0.16 |
| pul sys blood pressure 3 | 0.85 |
| pul dia blood pressure 3 | 0.74 |
| pul mean blood pressure 3 | 0.82 |
| art sys blood pressure 3 | 0.37 |
| art dia blood pressure 3 | 0.20 |
| art mean blood pressure 3 | 0.91 |
| systolic blood pressure ic | 0.54 |
| diastolic blood pressure i | 0.08 |
| fio2 1 | 0.30 |
| fio2 2 | 0.85 |
| fio2 3 | 0.69 |
| fio2 icu | 0.58 |
| paco2 1 | 0.30 |
| paco2 2 | 0.63 |
| paco2 icu | 0.69 |
| temp 1 | 0.84 |
| temp 2 | 0.45 |
| temp 3 | 0.38 |
| temp icu | 0.18 |
| cvp 1 | 0.79 |
| cvp 2 | 0.28 |
| cvp 3 | 0.76 |
| pasp 1 | 0.28 |
| pasp 2 | 0.24 |
| pasp 3 | 0.85 |
| padp 1 | 0.43 |
| padp 2 | 0.51 |
| padp 3 | 0.74 |
| cross clamp time | 0.51 |
| cpb time | 0.68 |
| creat1 | 0.38 |
| or fluid in | 0.78 |
| or fluid out | 0.05 |
| or net balance | 0.22 |
| rbc 1 | 0.28 |
| plt 1 | 0.43 |
| plt 2 | 0.34 |
| egfr 1 | 0.93 |
| hematocrit 1 | 0.62 |
| postop hr 1 | 0.58 |
| postop mabp 1 | 0.54 |
| postop temp 1 | 0.18 |
| postop mvo2 1 | 0.69 |
| uop 1 | 0.85 |
| uop 2 | 0.91 |
| uop 3 | 0.71 |
| uop 4 | 0.43 |
| uop 5 | 0.12 |
| uop 6 | 0.10 |
| uop 7 | 0.08 |
| age | 0.05 |
| smoking | 0.35 |
| body weight | 0.88 |
| Gender Male | 0.74 |
| BMI | 0.76 |
| CKD | 0.89 |
| Anemia | 0.28 |
| Beta Blocker | 0.20 |
| ACEI | 0.99 |
| Lowest Hct | 0.74 |
| Race White | 0.39 |
| Surgical category - combined | 0.82 |
| Surgical category - other | 0.99 |
| Surgical category - Valve | 0.46 |
| LVEF.PRE1.CATModerate | 0.60 |
| LVEF.PRE1.CATPoor | 1.00 |
| Pulmonary Hypertension | 0.75 |
| COPD | 0.95 |
| Unstable Angina | 0.99 |
| COPD Factor | 0.95 |
| LVFactor TRUE | 0.75 |
| Other Than CABG | 0.85 |
| Thoracic Aorta | 0.95 |
| Euro Score | 0.30 |
| Baseline eGFR | 0.42 |
| SV UP | 0.96 |
| SV DOWN | 0.99 |
| volume percent change | 0.64 |
| volume responsive | 0.72 |
| AKI Stage Stage 1 | 0.72 |
| ACE Inhibitors at first GFR | 0.99 |
| NSAIDS at first GFR | 0.83 |
| Antidiabetics at first GFR | 0.99 |
| log euro | 0.50 |
| Hypertension | 0.75 |
| Avg Lactic Acid | 0.17 |
| LV EA1 ratio | 0.15 |
| LV EA2 ratio | 0.15 |
| LV EA3 ratio | 0.79 |

P-values are from single-variable logistic regression models with 365-day GFR decline as the dependent (y) variable. In the case of categorical variables, each category is reported separately, but each category was a binary variable in a single logistic regression. P-values are from single-variable logistic regression models with 90-day GFR decline as the dependent (y) variable. In the case of categorical variables, each category is reported separately, but each category was a binary variable in a single logistic regression. AKI: acute kidney injury; LV: left ventricle; EDV: end diastolic volume; ESV: end systolic volume; SV: stroke volume; EF: ejection fraction; RV: right ventricle, CO: cardiac output; E: early diastolic peak velocity; A: late diastolic peak velocity; E/A ration of early to late peak diastolic velocities; DT: deceleration time; TAPSE: tricuspid annular plane systolic excursion; FIO2: fractional inspired oxygen concentration; PASP: pulmonary artery systolic pressure, PADP; pulmonary artery diastolic pressure; UOP: urinary output; temp: temperature; PaCO2:arterial partial pressure of carbon dioxide; PaO2: arterial partial pressure of oxygen; ACEI: angiotensin converting enzyme inhibitors; COPD: chronic obstructive pulmonary disease; CKD: chronic kidney disease; BMI: body mass index; eGFR: estimated glomerular filtration rate; ARBs: angiotensin receptor blockers; NASIDs: non-steroidal anti-inflammatory drugs; CABG: coronary artery bypass grafting; COPD: chronic obstructive pulmonary disease; Hct: hematocrit; CKD: chronic kidney disease; CVP: central venous pressure; RBCS: red blood cells; plts: platelets; FFP; fresh frozen plasma; LVEF.PRE1.CATModerate: moderate reduction in ejection fraction(EF 40-65%); LVEF.PRE1.CATPoor; severe reduction in EF (EF < 40%), ICU; intensive care unit; SV UP/DOWN: stroke volume with head up and down, respectively; eGFR: estimated glomerular filtration rate; in all, numerals,: time points for measurements.
